# Measuring the Inter and Intraspecific Sexual Shape Dimorphism and Body Shape Variation in Generalist Ground Beetles in Russia

**DOI:** 10.3390/insects11060361

**Published:** 2020-06-10

**Authors:** Hugo A. Benítez, Raisa A. Sukhodolskaya, Rodrigo Órdenes-Clavería, Tamara A. Avtaeva, Shapaat A. Kushalieva, Anatoly A. Saveliev

**Affiliations:** 1 Laboratorio de Ecología y Morfometría Evolutiva, Centro de Investigación de Estudios Avanzados del Maule, Universidad Católica del Maule, Talca 3466706, Chile; rodrigo.ordenescl@gmail.com; 2 Institute of Ecology and Mineral Resource Management Academy of Sciences of Tatarstan Republic, Tatarstan, Kazan 420000, Russia; sukhodolskayaraisa@gmail.com; 3 Kh. Ibragimov Complex Institute of the Russian Academy of Sciences, Grozny 364014, Russia; avtaeva1971@mail.ru; 4 Department of Biology and Methods of Teaching (Head), Chechen State Pedagogical University, Grozny 364014, Russia; hemiptera2013@mail.ru; 5 Department of Ecosystem Modeling, Kazan (Volga Region) Federal University, Kazan 420000, Russia; anatoly.saveliev.aka.saa@gmail.com

**Keywords:** geometric morphometrics, *Pterostichus*, *Carabus*, sexual size dimorphism, allometry

## Abstract

Ground beetles in multiple species vary greatly in the expression of the shape on sexual traits, resulting in a sexual shape dimorphism as a consequence of sexual selection differences. The present research focuses on the study of inter and intrasexual sexual shape dimorphism of two generalist genera of ground beetles *Pterostichus* and *Carabus*. Geometric morphometric methods were applied to five generalist species of ground beetles *Carabus exaratus*, *C. granulatus*, *Pterostichus melanarius*, *P. niger*, and *P. oblongopunctatus* and several multivariate analyses were applied for two different traits, abdomen and elytra. Three of the five species analyzed showed high levels of sex-based shape dimorphism. However, the most generalist species, *P. melanarius* and *P. oblongopunctatus*, did not evidence shape-based sexual dimorphism differentiation in both of the analyzed traits, as statistically confirmed based on the permutation of pairwise comparison of the Mahalanobis distances of a sex–species classifier. It is generally known that environmental stress in natural populations can affect the fitness expression, principally related to sexual fecundity, being that this pattern is more evident in non-generalist species. In our results, the contrary pattern was found, with the absence of sexual shape dimorphism for two of the three generalist species analyzed. On the other hand, the interspecies shape variation was clearly identified using principal component analysis of both of the analyzed traits. Finally, this research is the first to analyze the relationship between sexual shape dimorphism in Russian ground beetles, evidencing the lack of understanding of the mechanism underlying the sexual dimorphism, especially in species living in extreme environments.

## 1. Introduction

The direction and degree of sexual differences in body shape vary greatly among animal taxa. This phenomenon has launched a large number of studies devoted to explaining the evolutionary mechanisms underlying among-species patterns of sexual dimorphism. Size and shape are defining trait measurements of all organisms, impacting a variety of basic functions, such as dispersal ability, intraspecific competition, and reproductive output [1,2,3,4,5]. These functions are in turn selected upon by natural and sexual selection; thus, studying the size and shape differences among and between species and individuals of a species can reveal important information about evolutionary pressures acting on that species [6]. Sexual size dimorphism (SSD) is defined as the significant differentiation between size traits, particularly the body size of a species, between males and females [7]. This phenomenon is mostly related to sex differences and their relationship between body size and fitness (fecundity and mating success). Mating success regarding sexual dimorphism has been shown to be under selection in numerous comparative studies [8,9,10,11,12]. 

SSD is an object widely studied presently in insects; in the majority of species, it is female-biased [13,14]. As size is a result of many underlying factors, proximate factors such as growth rates and energy requirements can inhibit a sex from reaching its theoretical size optimum [6]. On the contrary, male-biased SSD is considered to be one of the major determinants of mating success in many species [15,16,17,18]. Since bigger males are normally more aggressive and more competitive than smaller males, bigger males often attain greater reproductive success through intrasexual selection [6,19]. The direction and degree of sexual changes in size vary significantly among different animal taxa [17,20,21,22]. Contrary to SSD, sexual shape dimorphism (SShD) has received much less attention and is defined as the relationship in shape or form between males and females [7,23,24,25,26]. Studies in insects about SShD have discussed it as a diagnostic trait in ontogenetic analysis, particularly related to allometry and also to sex identification where the shape is the principal trait in the study [19,27,28,29,30,31]. In order to study SShD, geometric morphometric tools are able to estimate the association of shape and size related to the variance in males and females [31,32,33]. Using this tool, the shape of sexual traits can be studied to reveal patterns of disparity at different spatial and temporal scales [34].

In ground beetles, geometric morphometrics has been used to help in the identification of cryptic species [35,36], to assess ecomorphological differentiation [34,37,38,39,40,41], and likewise is an important tool to study the sexual dimorphism [25,31,42,43,44,45]. In other arthropods, geometric morphometrics is used to understand the shape variability in spiders belonging to urbanized territory [46,47]. Despite the number of articles published on insects to understand the sexual dimorphism using geometric morphometrics, there is still a significant amount unknown about the roles of the selection of the dimorphism regarding shape variation, which is principally related to extreme or inhospitable environments where the effort required to build field collections is proportionally minor compared to the effort required to collect insects in tropical areas. Therefore, the following research aimed to complement the information for ground beetles collected in less urbanized and inhospitable environments, which is most of the unexplored Russian territory, in order to understand and assess the relationship between the intra- and intersexual shape dimorphism between and within different groups of species.

## 2. Materials and Methods 

### 2.1. Sample Sites and Data Acquisition

Adult ground beetles were collected from different areas in Russia; the specimens collected belong to five species of two different genera [*Carabus* Linnaeus, 1758 and *Pterostichus* Bonelli, 1810 (Coleoptera, Carabidae)], *C. exaratus* Quensel, 1806, *C. granulatus* Linnaeus, 1758, *P. melanarius* (Illiger, 1798), *P. niger* (Schaller, 1783) and *P. oblongopunctatus* (Fabricius, 1787). Specimen of *C. exaratus* were sampled in the suburbs of Kenkhi village (North Caucasus highland in the Chechnya Republic at 1923 masl). The other four species were sampled at the state wildlife sanctuary Volzchskie prostori particularly from islands that are periodically flooded due to the changing water level in the Volga river. The islands’ air and soil temperatures change with higher amplitudes in the vegetation season and during the day/night period as well if compared with the mainland habitats.

### 2.2. Shape Analysis 

**Figure 1:**
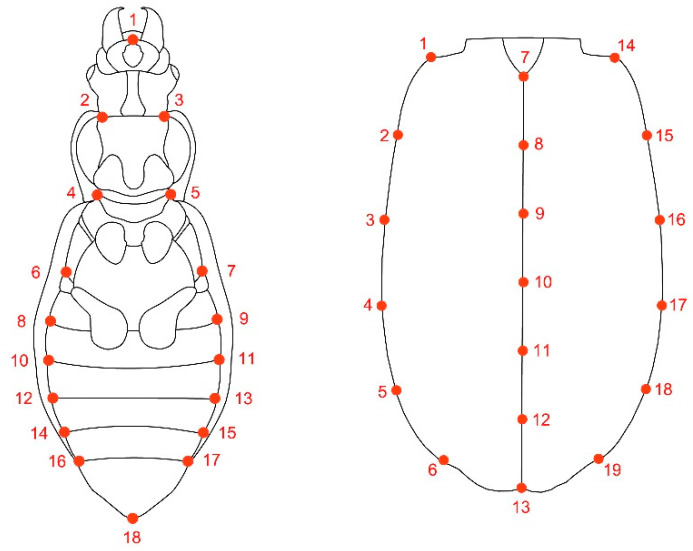
Graphical representation of the location of the 18 and 19 Landmarks (LMs) in ventral and elytral view for the different ground beetle species.

For the analysis, 100 specimens (20 specimens per species) were used. The sex was determined through the examination of the abdominal apex and forelegs, the latter being broadened in males. The analysis, which considered variation in shape exclusively, was performed on male and female specimens mounted in the dorsal and ventral position, digitized by a Nikon D5100 camera with a custom opaque light disperser and a box with an opaque reflective surface following the procedure for beetles in geometric morphometrics [25]. The 18 and 19 landmarks were digitized in the elytral and ventral views, respectively (LMs, anatomical homologous points), on every picture, using the software TpsDig2v.2.31 [48] (Figure 1). Landmark coordinates were obtained for all specimens after a Procrustes superimposition procedure, which removes the information of size, position, and orientation to standardize each specimen according to centroid size [49,50].

In order to avoid a measurement error in the landmarking procedure, a sample of individuals was digitized twice, and a Procrustes ANOVA was performed in order to compare whether the mean squares (MS) values for the individuals were lower than the error. This procedure denoted that there were no problems or landmarks misplaced [51,52]. The sexual shape dimorphism was evaluated using a principal component analysis (PCA) of the covariance matrix of the shape variation from the entire dataset [53]. In order to identify whether the allometry has some influence on the shape variation, multivariate regression was performed using the shape as a dependent variable and centroid size as an independent variable [54]. In order to assess statistically and graphically the differences in sexual shape dimorphism, a canonical variate analysis (CVA) was performed for the elytral and ventral view. A permutation test was performed with the Mahalanobis distances (morphological distances extracted after a CVA). 

Differences between species and sexual dimorphism were assessed, superimposing the average shape of the species and their sex, respectively. All analyses were then run using MorphoJ software version 1.05c [55].

## 3. Results

One of the main analyses in geometric morphometrics is the measurement error. With its results, it is possible to confirm that the landmarking process was in accord with the morphometric standards [52]. The Procrustes ANOVA for assessing the measurement error showed that the mean square for individual variation exceeded the measurement error in elytral and ventral views. This means no error in the landmarking procedure (Table 1). 

**Figure 2:**
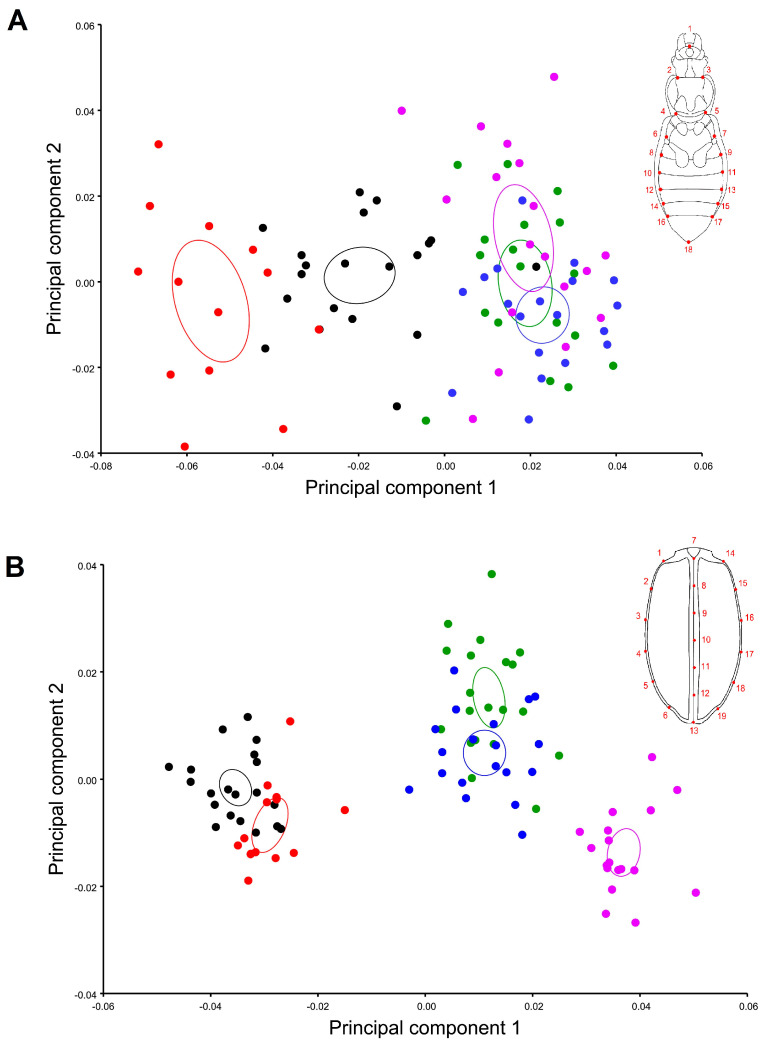
Principal component analysis of the ventral (**A**) and elytral (**B**) shape variation between multiple ground beetle species. The graphical visualization represents the shape space for the five species represented by colors: Red: *Carabus exaratus*, Black: *Carabus granulatus*, Green: *Pterostichus melanarius*, Blue: *Pterostichus niger*, Purple: *Pterostichus oblongopunctatus*. * Each point represents a different shape.

**Figure 3:**
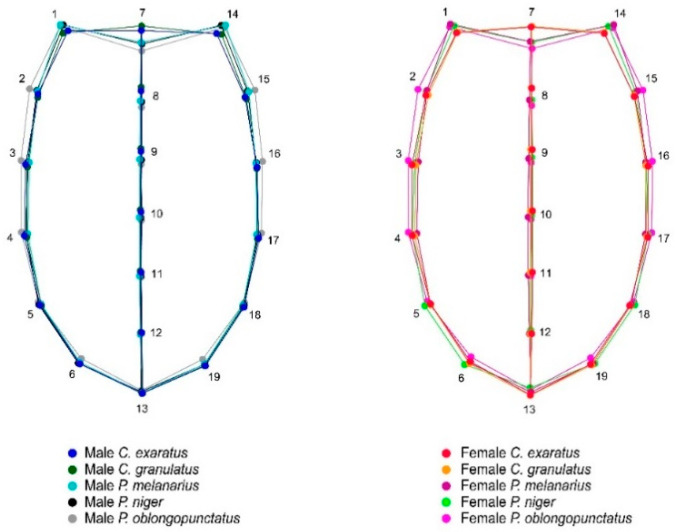
Wireframe representation of the superimposition of average elytral shapes and their corresponding landmarks between males and females in all the corresponding analyzed species in this study: *Carabus exaratus*, *Carabus granulatus*, *Pterostichus melanarius*, *Pterostichus niger*, and *Pterostichus oblongopunctatus*. * The wireframe was aligned using starting shape.

**Figure 4:**
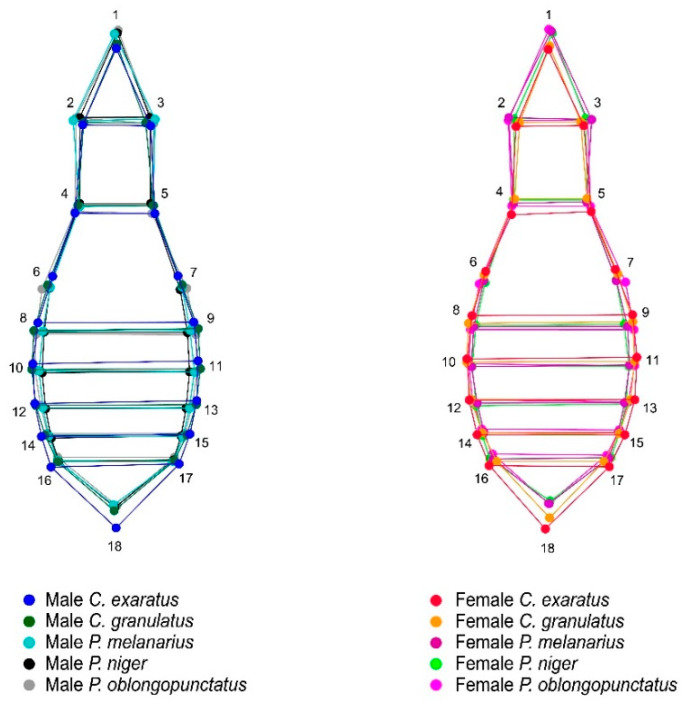
Wireframe representation of the average ventral shape variations and their corresponding landmarks between males and females of all the corresponding species analyzed in this study: *Carabus exaratus*, *Carabus granulatus*, *Pterostichus melanarius*, *Pterostichus niger*, and *Pterostichus oblongopunctatus*. * The wireframe was aligned using starting shape.

The PCA showed that the majority of the shape variation was explained in the first three dimensions for both views, accounting for 71% (PC1 = 43.7 %; PC2 = 14.7 %; PC3 = 13.3 %) of the total ventral shape variation (Figure 2A) and 87.03% (PC1 = 64.2 %; PC2 = 14.2 %; PC3 = 8.5 %) of the total shape variation (Figure 2B). Average shape variation between species was superimposed in order to analyze interspecific shape variation independently by sex in elytral and ventral view (Figure 3 and Figure 4).

**Figure 5:**
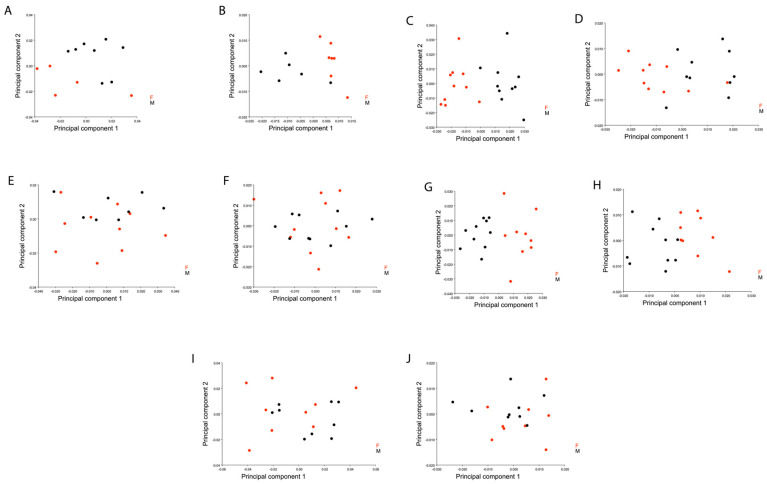
Principal component analysis to represent the intrasexual shape dimorphism between species, the left column (**A**,**C**,**E**,**G**,**I**) represent the ventral view and the right column (**B**,**D**,**F**,**H**,**J**) elytral view of *Carabus exaratus*, *Carabus granulatus*, *Pterostichus melanarius*, *Pterostichus niger*, and *Pterostichus oblongopunctatus*, correspondingly. The graphical visualization represents the shape space for the five species independently and red represents females and black represents males.

The higher percentage observed in both views may be attributable to the influence of centroid size on the shape (allometry), the multivariate regression showed 49% (*p*-value: <0.0001) of allometric influence on the elytral view and 27.2% (*p*-value: <0.0001) on the ventral view. After allometric correction, a PCA of the covariance matrix of the residual of the regression was used, the shape variation of both views decreased and changed considerably for the first three shape dimensions of the PCA; in the elytral view, it accounts for 75.8% (PC1 = 35.8 %; PC2 = 28.06 %; PC3 = 12.05 %) and in the ventral view, 65% (PC1 = 33.7 %; PC2 = 19.7 %; PC3 = 11.9 %). Sexual dimorphism was evident in three of the five species studied by means of independent PCA calculation for each species (Figure 5). The results showed that *P. melanarius* and *P. oblongopunctatus* were the only species where points became mixed between one another for dimensions one and two of the shape space (Figure 5E,F,I,J). 

**Figure 6:**
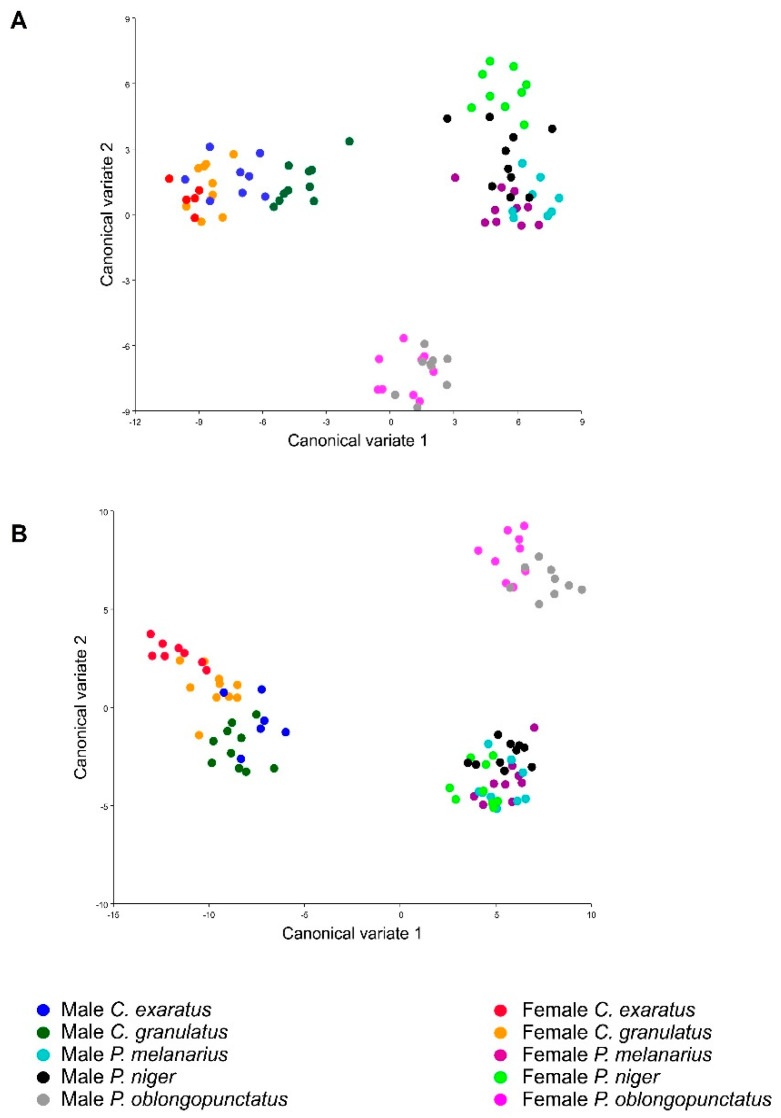
Canonical variate analysis of sexual shape dimorphism, using sex and species as a combined classifier (**A**): ventral view, (**B**): elytral view.

The CVA showed a clear separation between both genera and differences between sexual shape dimorphism regarding the analyzed view, with clearer differentiation in the ventral view, as the PCA showed, and less variation in the elytral view. Nevertheless, once the Mahalanobis distances were compared, statistical differences were found between a pairwise comparison of shape variation between species and sex (Figure 6, Table 2 and Table 3).

**Figure 7:**
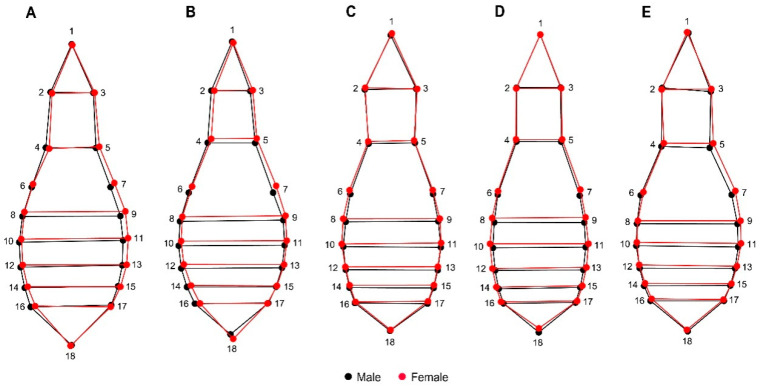
Wireframe representation of the superimposition between the average ventral shape between male and females, and their corresponding landmarks in all the analyzed species in this study. (**A**): *Carabus exaratus*, (**B**): *Carabus granulatus,* (**C**): *Pterostichus melanarius*, (**D**): *Pterostichus niger,* and (**E**): *Pterostichus oblongopunctatus*.

The average shape of the ventral view showed a noticeable expansion of the female abdomen, where, globally, for the five species, the landmarks 16, 17, and 18 show wider abdominal sternites, representing a robust abdomen, and, for males, the variation was more evident in the thoracic region, where the propisternum landmarks 4 and 5 denote a wider thorax than females (Figure 7).

**Figure 8:**
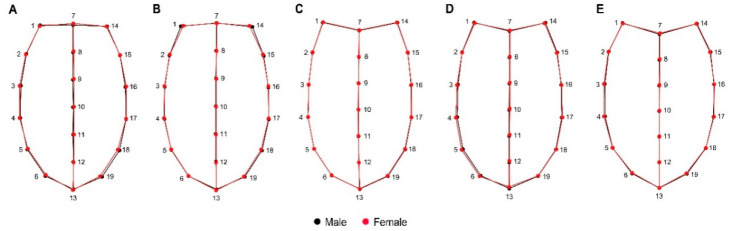
Wireframe representation of the superimposition between the average elytral shape between male and females, and their corresponding landmarks in all the analyzed species in this study. (**A**): *Carabus exaratus*, (**B**): *Carabus granulatus,* (**C**): *Pterostichus melanarius*, (**D**): *Pterostichus niger,* and (**E**): *Pterostichus oblongopunctatus*.

Particularly, the elytral view shows the morphometric variation in elytra, which can be seen entirely as a thicker and more robust structure in females and more slender and thinner in males. Landmark 7 represents the scutellum intersection between the right and left elytra, which is longer in females than in males (Figure 8). This relationship of size and shape is normally attributed to allometry; however, in both the elytral and ventral views, the aspects related to the centroid size of shape showed that females are clearly longer than males in the five species studied.

## 4. Discussion

Inter and intrasexual shape dimorphism differences were found in three of the five species studied using geometric morphometric techniques. Each of the three species in this research showed high levels of sex-based shape dimorphism. It is well known that environmental stress or perturbation in natural populations can affect the fitness expression or even the population sex ratio of the organism, reflecting this perturbation in morphological variability (e.g., Polak [56] developmental instability). The sampled specimens from across the different locations in the Russian highlands and the islands show particularly little perturbation in abundance. Nevertheless, the individuals of *Pterostichus* species (*P. melanarius* and *P. oblongopunctatus*), due to their generalist condition, seem to be more abundant than *C. granulatus* from the islands independently affected by occasional floods from the Volga river. This condition of the generalist species could benefit the adaptation of species to inhabit environments with multiple stressors, buffering some mechanisms of evolutionary plasticity which seems to be the case for *P. melanarius* and *P. oblongopunctatus.*

Another explanation of the absence of SShD was provided by Benitez et al. [19], testing the hypothesis that beetles with similar abundance or population sex ratios did not differ greatly in their pattern of SSD and SShD, confirming their research experimentally using a macroevolutionary Bayesian approach in the genus of ground beetle *Ceroglossus*. This evidence confirms that sexual fecundity may be directly related to SShD and sexual selection, driving the evolution of sexual dimorphism, being analyzed in some comparative studies [8,19,42,57,58]. 

For our results, the differentiation between males and female has achieved sex-specific phenotypic expression, particularly noticed in the ventral view (abdomen), although not evident in the elytral view corresponding to the elytral shape variation. The latter is a trait that changes interspecifically and is mostly used to identify groups of families or genera in beetles [59,60,61]. Contrary to the elytral shape variation, the abdominal shape between sexes was found to be directly influenced by sexual dimorphism at intraspecific levels in three of the analyzed species. The intraspecific SShD in the abdominal morphology showed that males tend to have a less robust abdomen than females; this is related to a wider proepisternum in males and wider abdominal sternites in females [19,42]. The other observed pattern in the results was the higher levels of allometry for both the elytral and ventral views; this phenomenon is directly associated with the SSD. Allometry is defined as the association between size and shape, or the covariation of parts due to a variation in size. It has been highly studied alongside SSD in insects [2,10,28,43,62,63,64,65,66] and particularly in beetles [24,28,67,68,69,70,71,72,73,74]. The type of allometry is classified according to the cause of variation; the inter- and intrasexual shape dimorphism found have a static allometric relationship (for adult insects). Nevertheless, this could also be associated with larval ontogenetic growth and nutritional causes.

## 5. Conclusions

To conclude, geometric morphometrics was a powerful tool to analyze the SShD at different species of generalist ground beetles, allowing us to detect a pattern of inter and intrasexual dimorphism. This is the first study to analyze the expression of dimorphism in shape variables in Russian beetles. Nonetheless, more studies are needed to understand the developmental pattern that causes the sexual shape dimorphism in ground beetles, insufficient knowledge within species-level variation is unfortunate per se, particularly for non-model species and even less for species inhabiting extreme environments, generating confusion on the understanding of evolutionary mechanisms behind the between-species patterns of sexual dimorphism. Consequently, the challenge is clear, a better understanding, increasing the research support for non-model species and, for extreme environmental species in evolutionary developmental biology urges to promote better hypotheses about the origins of sexual shape dimorphism in ground beetles.

## Figures and Tables

**Table 1:** Measurement error analysis by Procrustes ANOVA for both centroid size and body shape for elytra and ventral view in a subsample of the analyzed ground beetles.

**Centroid size**	**Elytra**				
Effect	SS	MS	df	F	*p*(param)
Individual	28.834975	1.517630	19	553	<0.0001
**Error 1**	0.054883	0.002744	20		
**Shape**					
Effect	SS	MS	df	F	*p*(param.)
Individual	0.01459804	0.0000225976	646	35.84	<0.0001
**Error 1**	0.00042873	0.0000006305	680		
**Centroid size**	**Ventral View**				
Effect	SS	MS	df	F	*p*(param)
Individual	47.130744	2.772397	17	20.45	<0.0001
**Error 1**	2.440455	0.135581	18		
**Shape**	SS	MS	df	F	*p*(param.)
Individual	0.03155336	0.0000580025	544	34.30	<0.0001
**Error 1**	0.00097397	0.0000016909	576		

**Table 2:** Mahalanobis distances and the respective *p*-values of the pairwise permutation, for the elytral view comparison of sexual shape dimorphism using sex and species as a combined classifier.

Elytral View Mahalanobis Distance									
Sex/Species	F/Ce	F/Cg	F/Pm	F/Pn	F/Po	M/Ce	M/Cg	M/Pm	M/Pn
F/Cg	5.2613								
*p*-value	<0.0001								
F/Pm	18.8655	16.2569							
*p*-value	<0.0001	<0.0001							
F/Pn	17.953	15.4154	5.7224						
*p*-value	<0.0001	<0.0001	00.0001						
F/Po	18.7788	17.0917	11.8677	12.4862					
*p*-value	<0.0001	<0.0001	<0.0001	00.0001					
M/Ce	7.6355	4.5361	14.2137	12.9436	16.0726				
*p*-value	<0.0001	00.0001	<0.0001	<0.0001	<0.0001				
M/Cg	9.1398	5.348	15.1158	14.1775	17.6648	4.9439			
*p*-value	<0.0001	00.0001	<0.0001	<0.0001	<0.0001	0.0002			
M/Pm	18.9741	16.2627	4.0372	6.3808	12.1244	13.9327	14.6692		
*p*-value	<0.0001	<0.0001	00.0001	<0.0001	<0.0001	<0.0001	<0.0001		
M/Pn	18.474	15.8697	4.3609	4.1095	10.5852	13.5252	14.6722	4.5779	
*p*-value	<0.0001	<0.0001	<0.0001	<0.0001	<0.0001	<0.0001	<0.0001	<0.0001	
M/Po	20.3573	18.5016	11.0318	11.8492	4.1636	17.0685	18.5699	11.0096	9.5144
*p*-value	<0.0001	<0.0001	<0.0001	<0.0001	0.001	<0.0001	<0.0001	<0.0001	<0.0001

**Table 3:** Mahalanobis distances and the respective *p*-values of the pairwise permutation for the ventral view in the comparison of sexual shape dimorphism using sex and species as a combined classifier.

Ventral View Mahalanobis’ Distance									
Sex/Species	F/Ce	F/Cg	F/Pm	F/Pn	F/Po	M/Ce	M/Cg	M/Pm	M/Pn
F/Cg	12.632								
*p*-value	0.0001								
F/Pm	16.19	16.2206							
*p*-value	0.0004	<0.0001							
F/Pn	17.0903	16.4136	7.5436						
*p*-value	0.0006	<0.0001	<0.0001						
F/Po	15.2336	13.855	10.1876	14.1468					
*p*-value	0.0001	<0.0001	<0.0001	<0.0001					
M/Ce	6.1634	14.2034	14.9344	15.6364	15.2469				
*p*-value	0.0009	0.0001	<0.0001	<0.0001	<0.0001				
M/Cg	12.5781	8.0316	12.5788	11.8953	11.6517	11.9983			
*p*-value	0.0007	<0.0001	<0.0001	<0.0001	<0.0001	0.0001			
M/Pm	17.3343	17.167	3.7356	6.6073	10.6839	15.9838	12.9631		
*p*-value	0.0002	<0.0001	0.0004	<0.0001	<0.0001	<0.0001	<0.0001		
M/Pn	17.7948	15.3642	7.5476	6.4857	11.7805	16.3224	11.0813	5.9446	
*p*-value	0.0002	<0.0001	<0.0001	<0.0001	<0.0001	<0.0001	<0.0001	<0.0001	
M/Po	15.6674	15.7041	10.0926	13.7685	5.2142	14.7927	11.6578	10.3142	11.4985
*p*-value	0.0003	<0.0001	<0.0001	<0.0001	<0.0001	0.0001	<0.0001	<0.0001	<0.0001
